# Multi-Omics Profiling Reveals Capsaicin Suppresses EBV Lytic Reactivation in Epithelial Cancers by Targeting Viral and Host Regulatory Networks

**DOI:** 10.3390/ijms27115146

**Published:** 2026-06-05

**Authors:** Nutchanat Chatchawankanpanich, Chanitchote Piyapittayanun, Chamsai Pientong, Chukkris Heawchaiyaphum

**Affiliations:** 1 Department of Biotechnology, Faculty of Science and Technology, Thammasat University, Pathumthani 12121, Thailand; nutchanat.c@dmsc.mail.go.th (N.C.); chanit@tu.ac.th (C.P.); 2 Department of Microbiology, Faculty of Medicine, Khon Kaen University, Khon Kaen 40002, Thailand; chapie@kku.ac.th; 3 HEC, Faculty of Medicine, Khon Kaen University, Khon Kaen 40002, Thailand

**Keywords:** capsaicin, EBV, host–virus interaction, viral reactivation, proteomics, metabolomics

## Abstract

Epstein–Barr virus (EBV) lytic reactivation contributes to the pathogenesis of EBV-associated epithelial malignancies, including nasopharyngeal carcinoma and gastric carcinoma, highlighting the need for therapeutic strategies targeting viral reactivation. Capsaicin exhibits anticancer and antiviral activities; however, its effects on EBV lytic reactivation remain unclear. This study investigated the effects of capsaicin on EBV lytic reactivation in EBV-positive epithelial cancer models. Capsaicin significantly suppressed the expression of lytic genes, including *BZLF1*, *BRLF1*, *BMRF1*, and *BLLF1*, and reduced EBV virion production. Proteomic analysis revealed alterations in host cellular pathways associated with metabolism, chromatin organization, and cytoskeletal regulation, whereas metabolomic profiling demonstrated perturbations in nucleotide, amino acid, and polyamine metabolism processes involved in viral DNA replication and protein synthesis. Protein–protein interaction network analysis identified key host proteins, including HSP90AB1, MYH9, and ANXA2, implicated in metabolic reprogramming, cytoskeletal organization, and stress responses. Moreover, upstream regulators associated with EBV lytic activation, including p65, AP-1, HIF-1α, and SP1, were down-regulated following capsaicin treatment. Collectively, these findings demonstrate a multitarget inhibitory effect of capsaicin on EBV lytic reactivation and support its therapeutic potential against EBV-associated epithelial malignancies.

## 1. Introduction

The Epstein–Barr virus (EBV) is a member of the gamma-herpesvirus subfamily with a 172 kb double-stranded DNA genome and is strongly associated with several malignancies, particularly epithelial cancers such as gastric cancer (GC) and nasopharyngeal carcinoma (NPC). Globally, more than 95% of adults are asymptomatically infected with EBV, highlighting its widespread prevalence. Transmission primarily occurs through bodily fluids, particularly saliva [1,2].

EBV infection alternates between latent and lytic phases, both of which contribute to viral persistence and disease pathogenesis. During latency, viral gene expression is highly restricted and the viral genome is maintained as an episome within the host nucleus [3]. Upon stimulation by chemical agents or other factors, EBV can be reactivated and enter the lytic cycle. Lytic gene expression occurs in a temporally regulated cascade and is classified into three groups: immediate-early, early, and late genes. The *BZLF1* and *BRLF1* genes encode the transcriptional activators Zta and Rta, respectively, which initiated the lytic cascade and subsequently induce the expression of early and late genes [2,3,4].

Accumulating evidence indicates that EBV lytic reactivation contributes to tumorigenesis [1,2,3,4]. Several EBV lytic gene products have been detected in the GC and NPC tissues [5,6]. Sero-epidemiological studies further demonstrate that elevated antibody titers against EBV lytic proteins, including DNA polymerase, and early antigen, are associated with an increased risk of GC, with detection rates ranging from 7.7% to 46.2% [6]. Similarly, antibodies against viral glycoproteins such as gp350 and gH/gL are linked to higher risk of NPC (OR = 2.27, 95% CI, 1.20–4.29, and OR = 2.18, 95% CI, 1.22–3.90, respectively) [7]. Furthermore, several EBV lytic proteins, including BARF1, BHRF1, BRLF1, BALF3, BGLF4, and DNase, have been shown to promote oncogenic processes such as genomic instability, inhibition of apoptosis, and enhanced cell proliferation [8,9,10]. Collectively, these findings highlight EBV lytic reactivation as a critical contributor to epithelial carcinogenesis and potential therapeutic target.

Although EBV-associated oncogenesis has traditionally been attributed to latent infection, increasing evidence supports a contributory role of the lytic cycle. Lytic infection promotes tumorigenesis through both cell-autonomous and non-cell-autonomous mechanisms, including the production of infectious virions and modulation of host oncogenic signaling pathways [11,12]. A key oncogenic mechanism mediated by EBV lytic proteins is the induction of genomic instability through disruption of the DNA damage response (DDR). BZLF1 impairs DNA double-strand break repair and disrupts cell cycle checkpoints, while early proteins can induce DDR markers such as pATM and γH2AX independently of viral replication, indicating their ability to trigger genotoxic stress [13]. Lytic replication further increases viral load and expands the population of infected cells susceptible to chromosomal alterations [2,14]. Single-cell transcriptomic study reveals heterogeneous reactivation states, including abortive and full lytic programs associated with activation of NF-κB, IRF3, and cellular reprogramming pathways [15].

Natural compounds have emerged as promising inhibitors of EBV lytic reactivation. Phytochemicals such as emodin, resveratrol, and luteolin suppress EBV virion production by inhibiting *BZLF1* and *BRLF1* expression and interfering with the transcription regulation [10,16,17,18,19]. Capsaicin (trans-8-methyl-N-vanillyl-6-nonenamide) is a bioactive phytochemical derived from *Capsicum* species, exhibits diverse pharmacological properties including anti-apoptotic, anti-inflammatory, antioxidant, metabolic-regulating, gastroprotective and anticarcinogenic properties [7,8,9,20,21,22,23,24,25,26,27]. In addition, capsaicin has demonstrated antiviral effects against several viruses, including herpes simplex virus, Lassa virus, vesicular stomatitis virus, encephalomyocarditis virus, and human influenza virus A virus [28,29,30]. However, its effects on EBV lytic reactivation and the underlying molecular mechanisms remain poorly understood, particularly in EBV-associated epithelial cancers such as GC and NPC.

In this study, we investigated the inhibitory effects of capsaicin on EBV lytic reactivation and its underlying mechanisms using integrated proteomic and metabolomic analyses. Capsaicin significantly suppressed chemically induced EBV lytic activation in EBV-positive epithelial cancer cells and modulated host signaling and metabolic pathways. Network and molecular docking analyses further suggested that capsaicin inhibits EBV reactivation through modulation of host regulatory proteins and potential direct interactions with viral lytic proteins, supporting its therapeutic potential in EBV-associated malignancies.

## 2. Results

### 2.1. Cytotoxicity of Capsaicin in EBV-Negative and EBV-Positive Epithelial Cancer Cells

To evaluate the inhibitory effects of capsaicin on EBV lytic reactivation, the cytotoxicity of capsaicin was first assessed in AGS-EBV, HONE1-EBV, and AGS cells using a CCK-8 assay kit. Capsaicin significantly inhibited the growth of the AGS, AGS-EBV, and HONE1-EBV cell lines at concentrations of 200 and 400 µM (Figure S1). A dose-dependent reduction in cell viability was observed in capsaicin-treated cells. Furthermore, the concentrations of capsaicin required for a reduction in cell viability of 50% (CC_50_) and 25% (CC_25_) were calculated. The cytotoxic concentrations of capsaicin at CC_50_ and CC_25_ are shown in Table 1. The CC_50_ values were 284.96 ± 9.05 µM for AGS-EBV cells, 301.22 ± 1.76 µM for HONE1-EBV cells, and 279.52 ± 2.52 µM for AGS cells. The comparable CC_50_ values between AGS-EBV and AGS cells indicate that EBV infection does not substantially alter the cytotoxic sensitivity of GC cells to capsaicin, suggesting that the antiviral effects observed in EBV-positive cells are not attributable to differential cytotoxic responses. Accordingly, CC_50_ and CC_25_ were selected for subsequent experiments as the upper boundary of the tested concentration range, with the recognition that CC_50_ represents a partially cytotoxic concentration at which 50% of cells remain viable, whereas CC_25_ more closely approximates a minimally cytotoxic condition.

### 2.2. Capsaicin Inhibits EBV Lytic Reactivation in EBV-Positive Epithelial Cancer Cells

**Figure 1:**
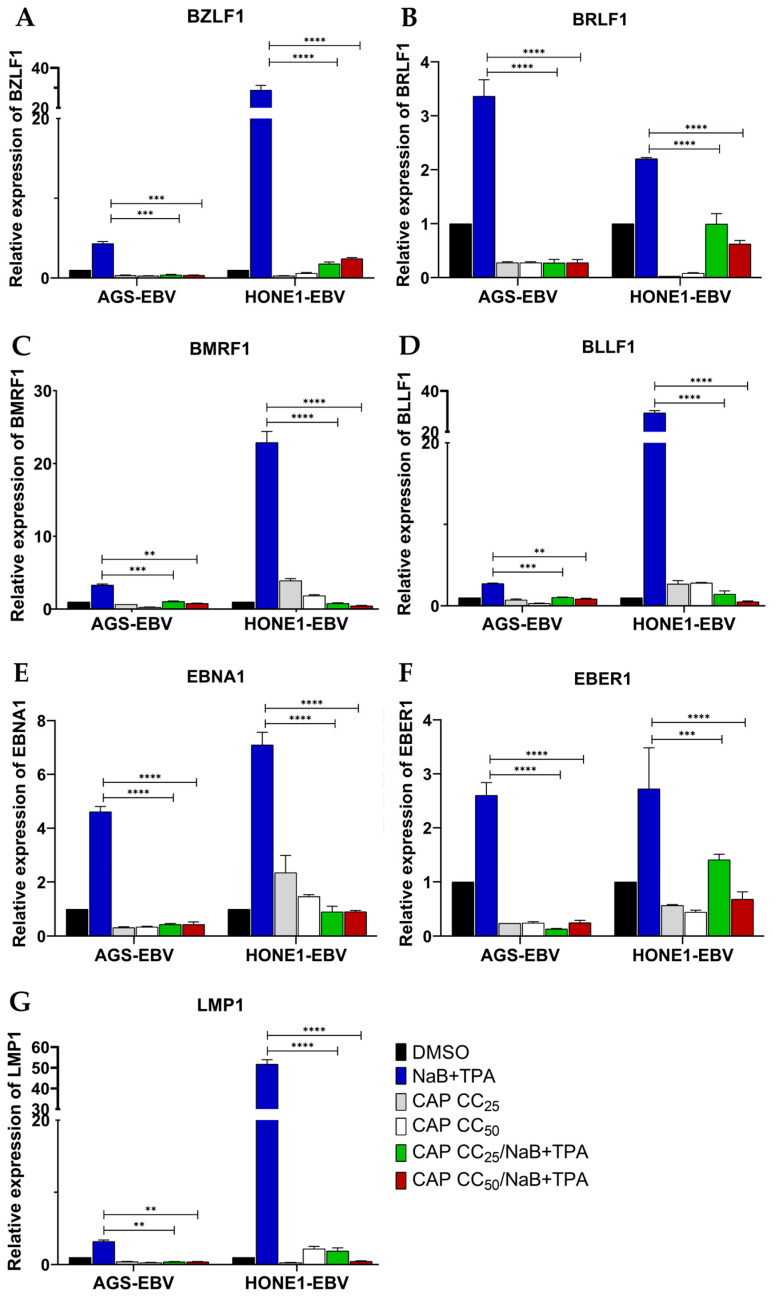
Effects of capsaicin on EBV gene expression in AGS-EBV and HONE1-EBV cells. Cells were pretreated with or without capsaicin for 3 h, followed by stimulation with NaB and TPA, and were further incubated for 48 h. The expression levels of EBV lytic genes, including *BZLF1* (**A**), *BRLF1* (**B**), *BMRF1* (**C**), and *BLLF1* (**D**), as well as EBV latent genes, including *LMP1* (**E**), *EBNA1* (**F**), and *EBER1* (**G**), were quantified by qRT-PCR. NaB, sodium butyrate; TPA, 12-O-tetradecanoylphorbol-13-acetate; CAP, capsaicin. ** *p* < 0.01, *** *p* < 0.001, **** *p* < 0.0001.

To determine whether capsaicin inhibits the lytic reactivation of EBV, EBV-positive cell lines were pretreated with capsaicin or DMSO for 3 h, followed by induction with the lytic inducers sodium butyrate (NaB) and 12-O-tetradecanoylphorbol-13-acetate (TPA). EBV gene expression was subsequently analyzed by quantitative real-time PCR (qRT-PCR). As expected, NaB and TPA treatment markedly induced the expression of the EBV lytic genes *BZLF1*, *BRLF1*, *BMRF1*, and *BLLF1*, in AGS-EBV and HONE1-EBV cells compared with the DMSO control (Figure 1). In contrast, co-treatment of capsaicin with NaB and TPA significantly reduced the expression of *BZLF1* and *BRLF1*, *BMRF1*, and *BLLF1* (Figure 1A–D). Additionally, capsaicin treatment significantly decreased the expression of the latent genes, *LMP1*, *EBNA1* and *EBER1* (Figure 1E–G). Thus, these findings indicate that capsaicin effectively suppresses EBV lytic reactivation, at least in part, through the downregulation of viral genes essential for lytic progression.

### 2.3. Capsaicin Inhibits EBV Virion Production in EBV-Positive Epithelial Cancer Cells

**Figure 2:**
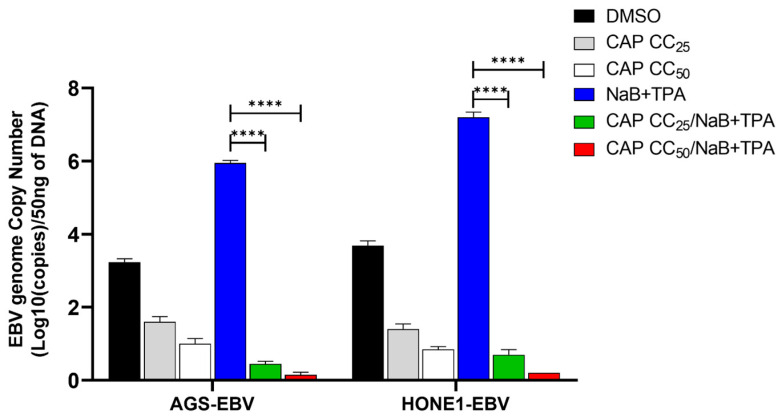
Capsaicin reduces EBV genome copy number in EBV-associated epithelial cancer cells. Cells were treated with DMSO (control), NaB/TPA, capsaicin, or a combination of capsaicin and NaB/TPA for 48 h. The EBV genome copy number in culture supernatants was quantified by qPCR. NaB, sodium butyrate; TPA, 12-O-tetradecanoylphorbol-13-acetate; CAP, capsaicin. ****: *p* < 0.0001.

The preceding results demonstrated that capsaicin suppresses EBV lytic reactivation by repressing viral gene expression. To further evaluate its effects on infectious progeny release, we examined EBV virion production in EBV-positive cell lines. Cells were treated with NaB/TPA alone, capsaicin alone, or capsaicin in combination with NaB/TPA, and the EBV genome copy number in the culture supernatants was quantified by qPCR. Consistent with the viral gene expression patterns, the EBV genome copy number was significantly reduced in cells treated with capsaicin alone and those treated with capsaicin in combination with NaB/TPA compared with cells subjected to NaB/TPA treatment alone (Figure 2). Consequently, these findings indicate that capsaicin effectively inhibits EBV virion production in EBV-positive epithelial cancer cells, primarily by suppressing the EBV lytic gene expression program required for mature virion assembly and release.

### 2.4. Selective Index of Capsaicin in Inhibiting EBV Lytic Reactivation

To assess the therapeutic selectivity of capsaicin against EBV lytic reactivation, the selectivity index (SI) was calculated by comparing its cytotoxicity to its antiviral efficacy. The cytotoxicity of capsaicin on EBV-positive cells was previously determined using a CCK-8 assay to obtain the CC_50_ concentration, whereas antiviral activity was assessed by quantifying EBV genome copy numbers following induction with NaB/TPA to determine the 50% inhibitory concentration (IC_50_). Capsaicin exhibited dose-dependent suppression of EBV virion production, with IC_50_ values falling below the CC_25_ threshold in both cell lines, suggesting that meaningful antiviral activity occurs at concentrations where cytotoxic effects are substantially lower than those observed at CC_50_ (Figure S2). The IC_50_ values were 79.29 ± 3.56 µM in AGS-EBV cells and 92.28 ± 4.24 µM in HONE1-EBV cells (Table 2). Based on the corresponding CC_50_ values, the SI (CC_50_/IC_50_) values were 3.60 for AGS-EBV cells and 3.26 for HONE1-EBV cells (Table 2). Consequently, these findings indicate that capsaicin preferentially inhibits EBV lytic reactivation rather than inducing nonspecific cytotoxicity, suggesting that it exerts antiviral activity within a therapeutically relevant concentration range.

### 2.5. Capsaicin Mediates Proteomic and Pathway Changes Underlying EBV Inhibition

To investigate the molecular mechanisms underlying capsaicin-mediated inhibition of EBV lytic reactivation, AGS-EBV and HONE1-EBV cells were pretreated with capsaicin prior to lytic induction with NaB/TPA. Total proteins were extracted and analyzed by LC-MS/MS-based proteomics. In total, 2960 proteins were identified in AGS-EBV cells, and 4840 proteins were identified in HONE1-EBV cells across all treatment conditions.

**Figure 3:**
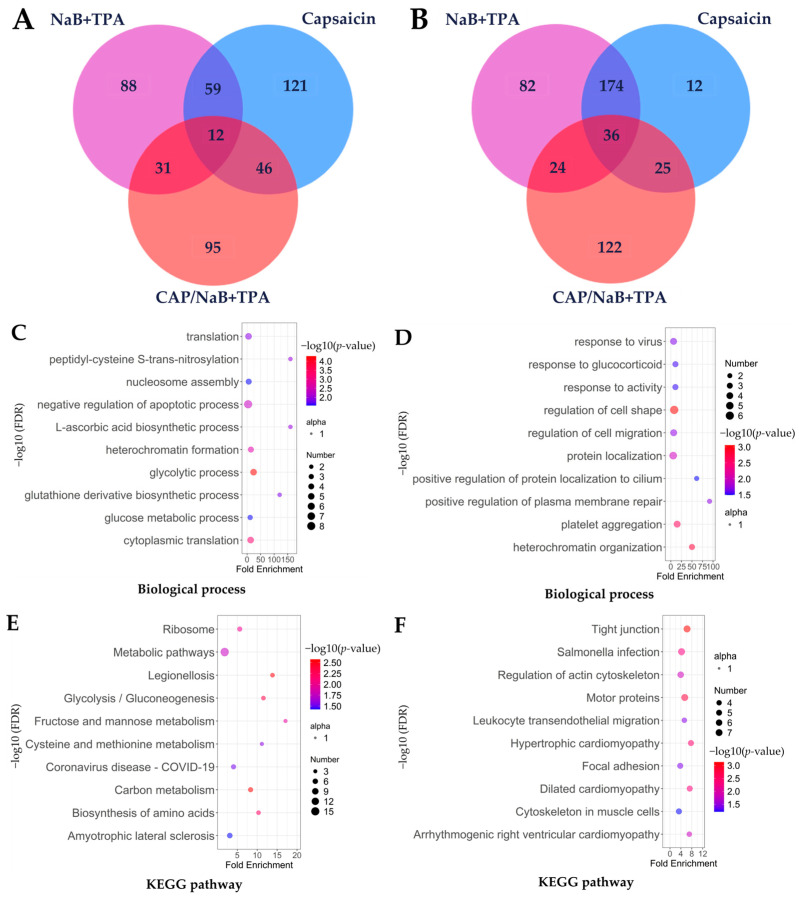
Proteomic and pathway analysis of capsaicin-mediated inhibition of EBV lytic reactivation in AGS-EBV cells. The DEPs were identified following treatment with capsaicin in combination with NaB/TPA. Venn diagrams illustrate the distribution of up-regulated (**A**) and down-regulated (**B**) proteins. The top 10 enriched BP terms are shown for up-regulated (**C**) and down-regulated (**D**) proteins. The top 10 enriched KEGG pathways for up-regulated (**E**) and down-regulated (**F**) proteins are also presented. NaB, sodium butyrate; TPA, 12-O-tetradecanoylphorbol-13-acetate; CAP, capsaicin.

Differential expression analysis revealed that, in AGS-EBV cells, treatment with capsaicin in combination with NaB/TPA resulted in 95 uniquely up-regulated proteins and 122 uniquely down-regulated proteins (Figure 3A,B). To further elucidate the biological significance of these differentially expressed proteins (DEPs), gene ontology (GO) and pathway enrichment analyses were performed. In terms of biological process (BP), up-regulated DEPs were significantly enriched in the glycolytic process, cytoplasmic translation, and negative regulation of the apoptotic process (Figure 3C), whereas down-regulated DEPs were predominantly associated with the regulation of cell shape, heterochromatin organization, and platelet aggregation (Figure 3D). Similarly, cellular component (CC) analysis indicated that up-regulated DEPs were primarily localized to the extracellular exosome, ribonucleoprotein complex, and nucleus (Figure S3A), while down-regulated proteins were principally associated with the extracellular exosome, brush border, and cytoplasm (Figure S3B). Likewise, molecular function (MF) analysis revealed that up-regulated proteins exhibited significant activities related to RNA binding, structural constituents of chromatin, and ubiquitin protein ligase binding (Figure S3C), whereas down-regulated proteins were linked to RNA binding, S100 protein binding, and identical protein binding (Figure S3D). Furthermore, KEGG pathway analysis further showed that up-regulated proteins participated in critical signaling pathways, including carbon metabolism, legionellosis, and glycolysis/gluconeogenesis (Figure 3E), while down-regulated proteins were predominantly involved in tight junctions, motor proteins, and hypertrophic cardiomyopathy (Figure 3F).

**Figure 4:**
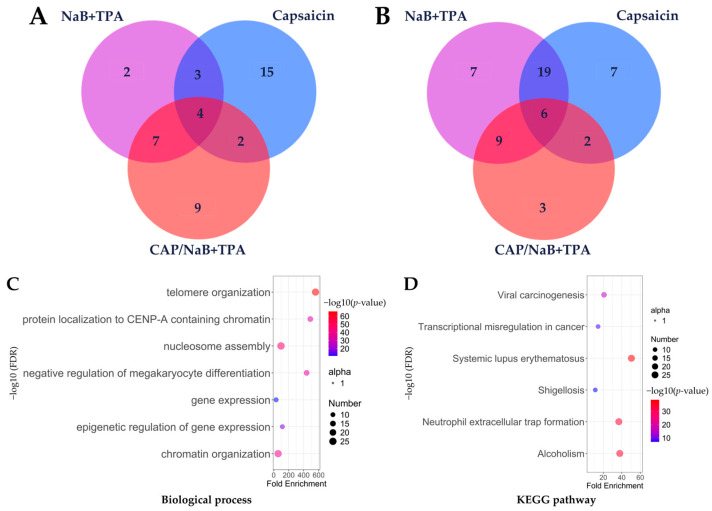
Proteomic and pathway analysis of capsaicin-mediated inhibition of EBV lytic reactivation in HONE1-EBV cells. The DEPs were identified following treatment with capsaicin in combination with NaB/TPA. Venn diagrams illustrate the distribution of up-regulated (**A**) and down-regulated (**B**) proteins. The top 10 enriched BP terms (**C**) and KEGG pathways (**D**) are shown for up-regulated proteins. NaB, sodium butyrate; TPA, 12-O-tetradecanoylphorbol-13-acetate; CAP, capsaicin.

In contrast, HONE1-EBV cells exhibited a markedly smaller number of DEPs, with only 9 uniquely up-regulated and 3 uniquely down-regulated proteins under the combination treatment of capsaicin with NaB/TPA (Figure 4A,B). Functional enrichment analysis suggested that the up-regulated proteins were primarily associated with telomere organization, nucleosome assembly, and chromatin organization (Figure 4C). In terms of CC, these proteins were mainly localized to the nucleosome, CENP-A-containing nucleosome, and protein-containing complexes (Figure S4A), whereas down-regulated proteins were associated with the extracellular exosome, vesicles, and cytosol (Figure S4B). In terms of MF, up-regulated proteins exhibited significant activities related to structural constituents of chromatin, protein heterodimerization activity, and DNA binding (Figure S4C), while down-regulated proteins were significantly involved in cadherin binding (Figure S4D). Additionally, KEGG pathway analysis showed that up-regulated proteins were enriched in pathways including systemic lupus erythematosus, alcoholism, and neutrophil extracellular trap formation (Figure 4D).

Collectively, these enrichment analyses demonstrate that capsaicin may influence multiple host cellular pathways, including metabolic processes, chromatin organization, and cytoskeletal regulation, thereby contributing to the inhibition of EBV lytic reactivation and virion production in a cell-type-dependent manner. However, these findings should be interpreted cautiously in HONE1-EBV cells due to the limited number of identified differentially expressed proteins.

### 2.6. Capsaicin-Mediated Metabolomic Reprogramming Underlying Inhibition of EBV Lytic Reactivation

**Figure 5:**
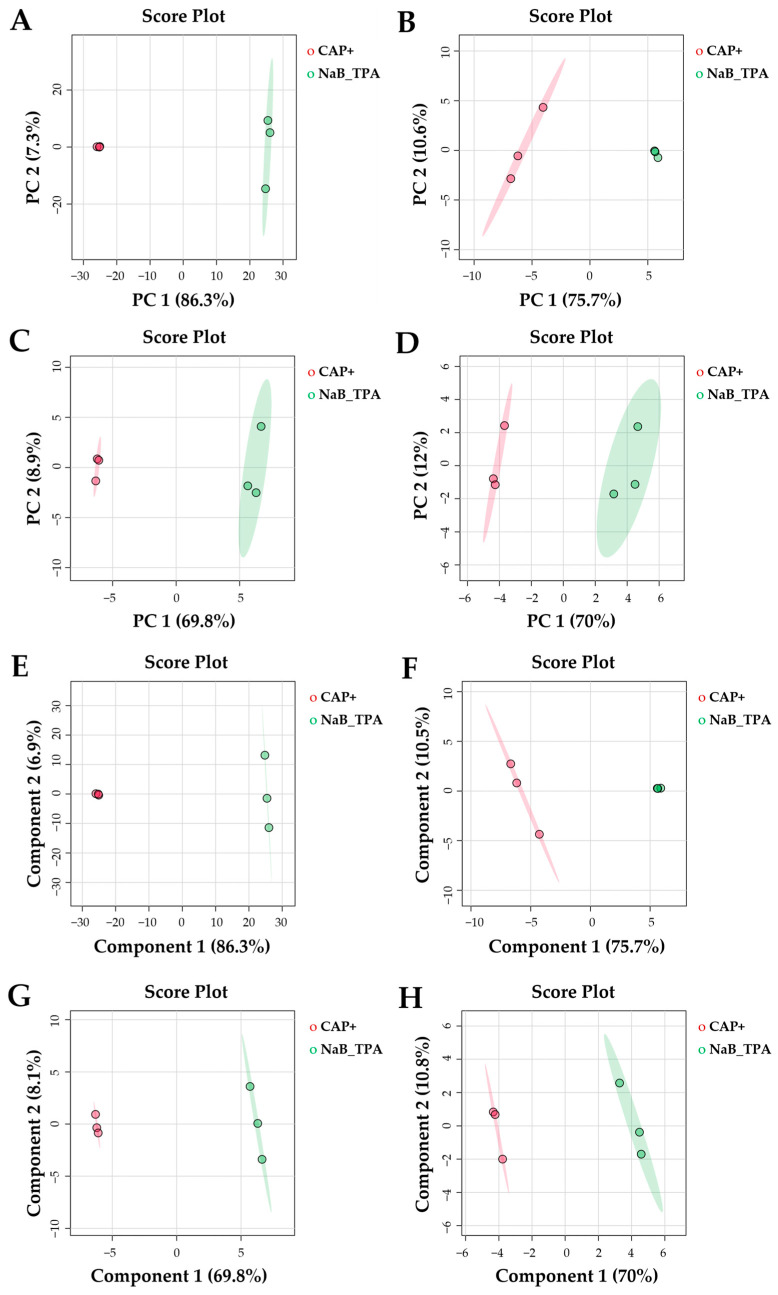
Capsaicin induces distinct metabolic profiles during the inhibition of EBV lytic reactivation. PCA and PLS-DA analyses of metabolic profiles in AGS-EBV and HONE1-EBV cells treated with NaB/TPA alone or in combination with capsaicin. PCA score plots of AGS-EBV cells are shown in positive (**A**) and negative (**B**) ionization modes, while those of HONE1-EBV cells are presented in positive (**C**) and negative (**D**) modes. PLS-DA score plots of AGS-EBV cells are displayed in positive (**E**) and negative (**F**) modes, whereas those of HONE1-EBV cells are shown in positive (**G**) and negative (**H**) modes. NaB, sodium butyrate; TPA, 12-O-tetradecanoylphorbol-13-acetate; CAP, capsaicin.

To further characterize the metabolic alterations associated with capsaicin-mediated suppression of EBV lytic reactivation, untargeted LC-MS-based metabolomic profiling was performed. As shown in Figure 5, multivariate analysis revealed distinct metabolic profiles among the treatment groups. Specifically, principal component analysis (PCA) demonstrated clear separation between the NaB/TPA-treated cells and those co-treated with capsaicin in both ionization modes. In AGS-EBV cells, PCA score plots showed pronounced separation along PC1 in both positive and negative modes (Figure 5A,B), indicating substantial metabolic reprogramming following capsaicin co-treatment. Similarly, HONE1-EBV cells exhibited distinguishable clustering between treatment groups in both positive and negative modes (Figure 5C,D), although the degree of separation was less pronounced compared with that of the AGS-EBV cells. Partial least squares discriminant analysis (PLS-DA) further demonstrated robust discrimination between treatment groups in both cell lines (Figure 5E–H). To confirm model reliability and exclude overfitting, five-fold cross-validation was performed for all PLS-DA models. In AGS-EBV cells, cross-validation yielded accuracy of 1.0, R^2^ values of 0.991–0.996, and Q^2^ values of 0.827–0.954 in positive and negative modes, respectively (Figure S5A,B; Table S1). In HONE1-EBV cells, similarly high performance was observed, with an accuracy of 1.0, R^2^ values of 0.996–0.999, and Q^2^ values of 0.861–0.988 across both ionization modes (Figure S5C,D; Table S1), thereby confirming the robustness of group discrimination in both cell lines.

**Figure 6:**
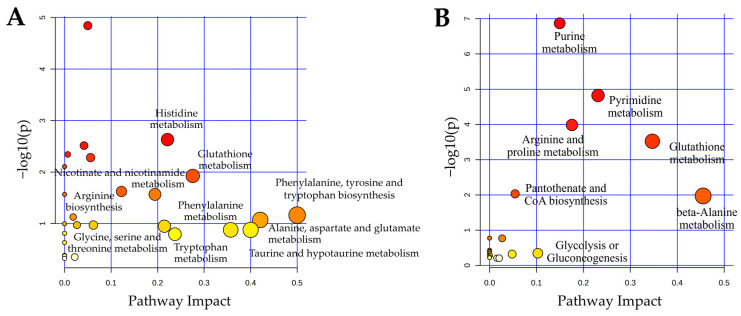
Capsaicin induces distinct metabolic pathway alterations during the inhibition of EBV lytic reactivation. Metabolic pathway analysis of differential metabolites in AGS-EBV (**A**) and HONE1-EBV (**B**) cells treated with NaB/TPA alone or in combination with capsaicin. The horizontal axis represents pathway impact values, while the vertical axis indicates statistical significance expressed as −log_10_(*p*-value). The color of each bubble indicates the *p*-value, with red representing higher statistical significance and yellow indicating lower significance. The size of each bubble reflects the pathway impact score, where larger bubbles correspond to higher impact values. Pathways meeting the criteria (impact ≥ 0.02 and *p* < 0.05) are considered significantly enriched.

Differential metabolite analysis (adjusted *p* < 0.05, VIP ≥ 1.0) identified significant alterations in response to capsaicin during EBV lytic induction. The PLS-DA models demonstrated satisfactory cross-validation performance, supporting the reliability of group discrimination. In AGS-EBV cells, capsaicin co-treatment with NaB/TPA significantly affected metabolites associated with energy metabolism, nucleotide biosynthesis, and amino acid metabolism (Figure 6A). Pathway enrichment analysis further highlighted purine metabolism, histidine metabolism, glutathione metabolism, nicotinate/nicotinamide metabolism, arginine biosynthesis, and phenylalanine-related pathways, indicating the coordinated modulation of nucleotide metabolism, redox homeostasis, and intermediary metabolism. In contrast, HONE1-EBV cells exhibited fewer metabolic alterations (Figure 6B), consistent with the more limited proteomic response observed in this cell line. Nevertheless, key pathways—including purine and pyrimidine metabolism, arginine and proline metabolism, glutathione metabolism, and β-alanine metabolism—were significantly affected, suggesting a more restricted modulation of nucleotide synthesis and oxidative stress-related pathways. Taken together, these findings demonstrate that capsaicin induces distinct metabolic alterations during EBV lytic reactivation, exerting its effects through the modulation of nucleotide biosynthesis, redox regulation, and amino acid metabolism.

### 2.7. Integrated Multi-Omics Analysis of Capsaicin-Mediated Suppression of EBV Lytic Reactivation

**Figure 7:**
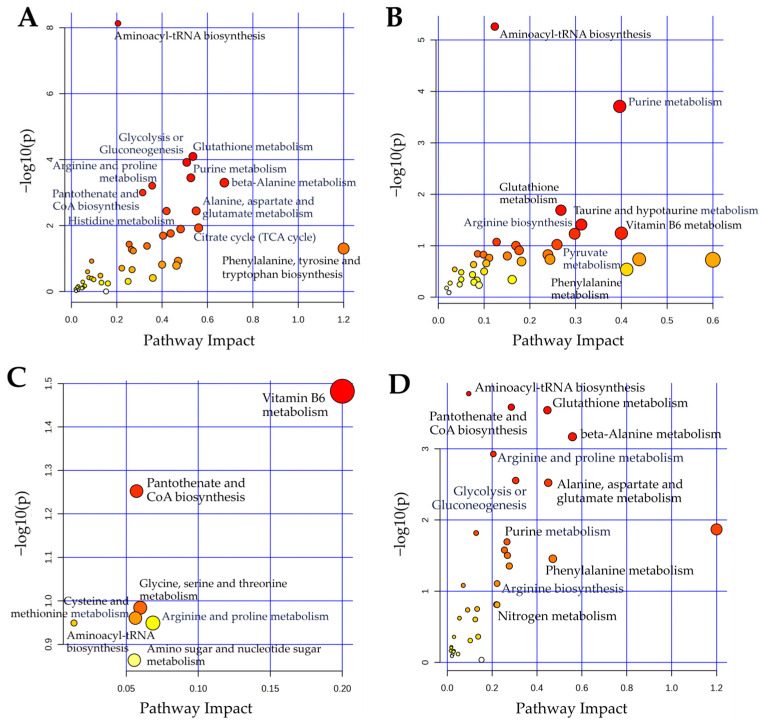
Integrated multi-omics pathway analysis in EBV-positive epithelial cancer cells. Joint pathway analysis integrating DEPs and differential metabolites identified significantly perturbed metabolic pathways in AGS-EBV and HONE1-EBV cells. Bubble plots depict the top altered pathways in up-regulated AGS-EBV (**A**), up-regulated HONE1-EBV (**B**), down-regulated AGS-EBV (**C**), and down-regulated HONE1-EBV (**D**) cells. Bubble size and color represent pathway impact and statistical significance, respectively. The horizontal axis represents pathway impact, while the vertical axis indicates statistical significance expressed as −log_10_(*p*-value). The color of each bubble indicates the *p*-value, with red representing higher statistical significance and yellow indicating lower significance. The size of each bubble reflects the pathway impact score, where larger bubbles correspond to higher impact values. Pathways meeting the criteria (impact ≥ 0.02 and *p* < 0.05) are considered significantly enriched.

Our proteomic results revealed the modulation of key biological processes, including glycolysis, cytoplasmic translation, and chromatin organization, as well as pathways associated with cytoskeletal dynamics and cell–cell interactions (Figure 3 and Figure 4). Consistently, metabolomic profiling demonstrated significant alterations in metabolites and pathways related to energy metabolism, nucleotide biosynthesis, and amino acid metabolism, including purine metabolism and glutathione metabolism (Figure 6). Therefore, to comprehensively elucidate the molecular mechanisms underlying capsaicin-mediated inhibition of EBV lytic reactivation, proteomic and metabolomic datasets were integrated using MetaboAnalyst. As anticipated, joint pathway analysis revealed that aminoacyl-tRNA biosynthesis, purine metabolism, and glutathione metabolism were prominently enriched among the up-regulated pathways in both AGS-EBV and HONE1-EBV cells (Figure 7A,B). In contrast, among down-regulated pathways, vitamin B6 metabolism exhibited the greatest impact in AGS-EBV cells (Figure 7C), whereas aminoacyl-tRNA biosynthesis and glutathione metabolism were markedly altered in HONE1-EBV cells (Figure 7D). Thus, these results indicate that capsaicin predominantly affects metabolic pathways involved in protein synthesis, nucleotide metabolism, and redox regulation during EBV lytic reactivation.

### 2.8. Protein–Protein Interaction Network Construction and Hub Protein Identification

**Figure 8:**
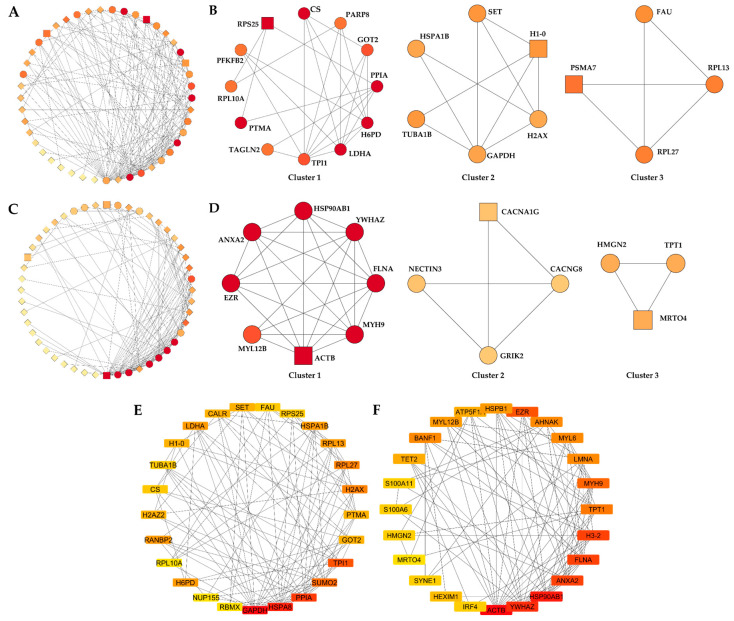
PPI network and hub protein analysis in AGS-EBV cells. The PPI network of up-regulated DEPs is shown in (**A**), followed by the corresponding highly interconnected cluster identified by MCODE analysis in (**B**), and the top 25 hub proteins ranked by degree value in (**C**). Similarly, the PPI network of down-regulated DEPs is shown in (**D**), the corresponding MCODE cluster in (**E**), and the top 25 hub proteins ranked by degree value in (**F**). Node color in the PPI networks reflects the degree of connectivity, ranging from light orange (low connectivity) to dark red (high connectivity), whereas square nodes indicate proteins with the highest degree values. In panels (**E**,**F**), the color gradient of protein labels represents their relative degree values, with darker red indicating higher network connectivity.

To identify key regulatory proteins involved in the capsaicin-mediated inhibition of EBV lytic reactivation, protein–protein interaction (PPI) networks were constructed based on the DEPs using the STRING database and visualized in Cytoscape software (version 3.10.4). The up-regulated PPI network comprised 45 nodes and 156 edges (Figure 8A), while the down-regulated network contained 48 nodes and 123 edges (Figure 8C). Both networks exhibited significant PPI enrichment (*p* < 1.0 × 10^−5^), indicating non-random and biologically meaningful interactions.

Subsequently, module analysis using the molecular complex detection (MCODE) algorithm identified the top three highly interconnected clusters in both up-regulated and down-regulated networks. In AGS-EBV cells, up-regulated clusters were primarily enriched in glycolysis, lactate metabolism, cytoplasmic translation, microtubule cytoskeleton organization, and nucleosome assembly (Figure 8B). In contrast, down-regulated clusters were associated with angiogenesis, platelet aggregation, the regulation of cell shape, calcium ion transmembrane transport, and RNA binding (Figure 8D). These clusters represent functionally coherent modules potentially involved in EBV lytic regulation.

Furthermore, hub protein analysis was performed using the CytoHubba plugin based on degree, maximal clique centrality (MCC), and betweenness centrality (BC) algorithms. The top-ranked hub proteins among up-regulated DEPs included TPI1, LDHA, H6PD, PPIA, GAPDH, CS, PARP8, H1-0, H2AX, and SET (Figure 8E and Table S2), whereas down-regulated hub proteins included MYH9, YWHAZ, FLNA, ANXA2, HSP90AB1, EZR, MYL12B, GRIK2, CACNG8, and NECTIN3 (Figure 8F and Table S2). These highly connected proteins were selected as candidate targets for further functional analysis.

**Figure 9:**
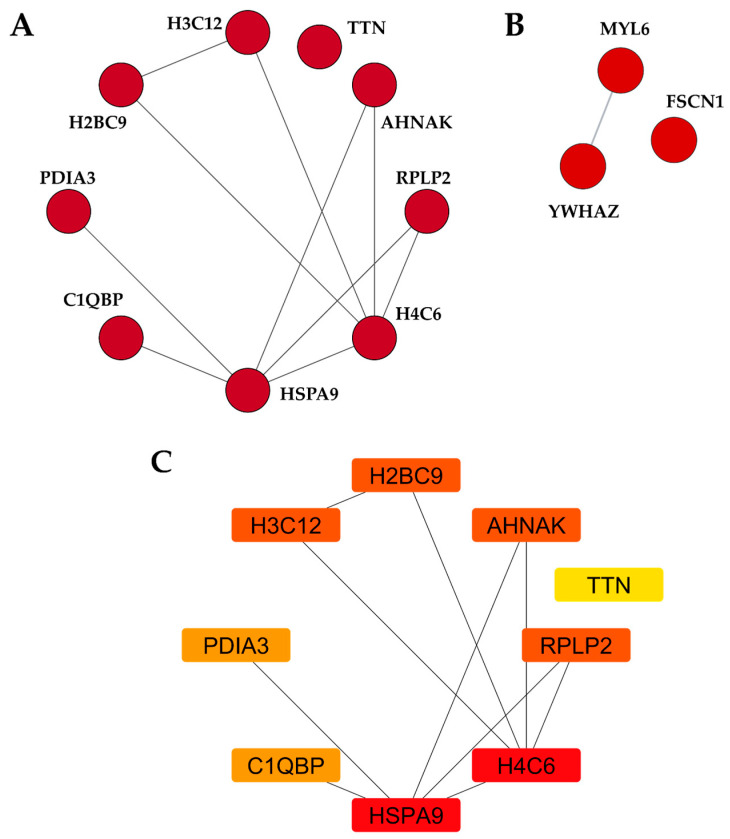
PPI network and hub protein analysis in HONE1-EBV cells. PPI networks of up-regulated (**A**) and down-regulated (**B**) DEPs were constructed. The top hub proteins from the up-regulated (**C**) network were identified based on degree values. Node color indicates degree of connectivity, dark red (high degree). In panels (**C**), the color gradient of protein labels reflects their relative degree values, with darker red indicating higher connectivity.

In contrast, in HONE1-EBV cells, MCODE-based module detection could not be reliably performed due to the limited number of DEPs and reduced network connectivity (Figure 9A,B and Table S3). Therefore, a hub-centric approach was applied. CytoHubba analysis identified nine up-regulated candidate proteins, including H4C6, HSPA9, H2BC9, RPLP2, H3C12, AHNAK, C1QBP, PDIA3, and TTN (Figure 9C and Table S3). For down-regulated DEPs, only three proteins (MYL6, YWHAZ, and FSCN1) were identified, reflecting insufficient network complexity for robust topological analysis.

Collectively, these results suggest cell-type-dependent differences in network complexity, with specific hub proteins and functional modules potentially associated with capsaicin-induced suppression of EBV lytic reactivation. However, the HONE1-EBV network analysis should be considered exploratory due to the limited number of DEPs and reduced network connectivity in this cell line.

### 2.9. Capsaicin Show Predicted Binding Interactions with Host Regulatory and EBV Lytic Proteins

**Figure 10:**
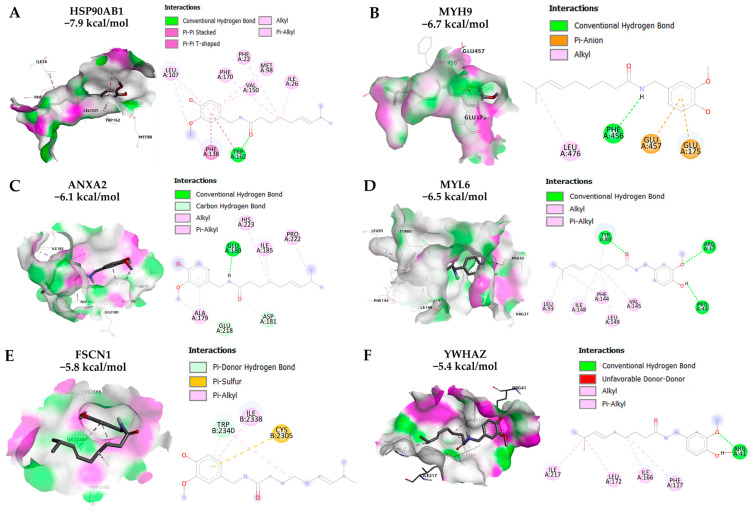
Capsaicin exhibits binding to host regulatory and EBV lytic proteins. Docking interactions of capsaicin with HSP90AB1 (**A**), MYH9 (**B**), and ANXA2 (**C**) in AGS-EBV cells, MYL6 (**D**), FSCN1 (**E**), and YWHAZ (**F**) in HONE1-EBV cells are shown. Each panel displays the three-dimensional binding pocket of the receptor with capsaicin (left) and the corresponding two-dimensional interaction map (right), with binding affinities expressed in kcal/mol.

Following identification of candidate hub proteins in AGS-EBV and HONE1-EBV cells, the top candidates were selected for molecular docking with capsaicin to computationally predict potential protein-ligand interactions computationally and estimate binding affinities as an exploratory analysis. Among the down-regulated hub proteins in AGS-EBV cells, HSP90AB1 showed the most favorable predicted binding affinity with capsaicin (−7.9 kcal/mol), with predicted interactions potentially stabilized by hydrogen bonding and π-π stacking with key residues Trp162 and Phe138 (Figure 10A). MYH9 and ANXA2 exhibited moderate-to-strong predicted binding affinities, with estimated binding energies of −6.7 and −6.1 kcal/mol, respectively (Figure 10B,C). The specific amino acid residues involved in these interactions are summarized in Table 1. Additionally, in HONE1-EBV cells, proteomic analysis identified a limited number of DEPs. For the limited number of down-regulated proteins identified in HONE1-EBV cells, MYL6, FSCN1, and YWHAZ showed predicted binding affinities of −6.5, −5.8, and −5.4 kcal/mol, respectively (Figure 10D–F), suggesting computational evidence of potential molecular interaction that warrants further experimental validation. The specific amino acid residues involved in capsaicin binding are summarized in Table 3.

To further elucidate upstream regulatory mechanisms, molecular docking was performed with key transcriptional regulators implicated in EBV lytic reactivation and cellular stress responses, including p65 (RELA), AP-1, STAT3, HIF-1α, SP1 and p53. Proteomic pathway analysis suggested suppression of signaling pathways associated with these transcriptional regulators were down-regulated following capsaicin co-treatment with NaB/TPA, consistent with potential suppression of signaling pathways associated with EBV lytic activation. In silico molecular docking simulations suggested that capsaicin may exhibit favorable predicted binding affinities with RELA (−5.9 kcal/mol) (Figure S6A). Notably, capsaicin showed strong predicted binding to STAT3 (−5.7 kcal/mol) and p53 (−5.2 kcal/mol), while moderate binding affinities were observed with AP-1 (−4.7 kcal/mol), HIF-1α (−4.7 kcal/mol), and SP1 (−4.4 kcal/mol) (Figure S6B–F). The specific amino acid residues involved in capsaicin binding are summarized in Table 4.

In addition, to assess whether capsaicin may directly target viral components, molecular docking was also performed with key EBV lytic proteins, including Zta (BZLF1), Rta (BRLF1), and early antigen-D (EA-D). Complementary experimental observations suggested reduced EBV lytic activation following capsaicin co-treatment. Docking simulations predicted that capsaicin may be accommodated within binding pockets of Zta, Rta, and EA-D, with computationally estimated binding energies of −4.5, −5.0 and −6.6 kcal/mol, respectively (Figure S6G–I and Table 4). The predicted interactions are suggested to be potentially stabilized by hydrogen bonding and hydrophobic contact with residues located within functionally relevant regions of the lytic proteins, although experimental validation will be required to confirm these interactions.

Collectively, these computational results suggest the possibility of capsaicin interactions with host hub proteins, transcriptional regulators, and EBV lytic proteins. However, these predictions require experimental confirmation by experimental assays before direct molecular interaction can be established. Taken together with proteomic evidence, these findings provide preliminary computational support for a multi-level mechanism in which capsaicin may inhibit EBV lytic reactivation through modulation of host signaling pathways and predicted interactions with viral protein function.

## 3. Discussion

EBV lytic reactivation plays a pivotal role in viral propagation and contributes to the pathogenesis of EBV-associated epithelial malignancies, including GC and NPC. Therefore, strategies that suppress the latency-to-lytic switch have emerged as promising approaches to limit viral spread and reduce EBV-driven oncogenic processes. To our knowledge, this study is the first to demonstrate that capsaicin effectively inhibits chemically induced EBV lytic reactivation in EBV-positive GC and NPC cells. By integrating proteomic, metabolomic, network, and molecular docking analyses, our findings provide mechanistic insights into how capsaicin disrupts host cellular programs required for efficient EBV lytic replication.

In the present study, cytotoxicity was first assessed to determine the tested concentrations (CC_25_ and CC_50_), thereby minimizing the amount of capsaicin used. These findings are consistent with previous reports demonstrating the anticancer efficacy of capsaicin in various malignancies, including prostate, lung, oral, osteosarcoma, colorectal, and gastric cancers [31,32,33,34,35,36,37,38,39], where CC_50_ values typically range from 50 to over 300 µM. Although these concentrations are effective for inducing apoptosis and cell cycle arrest in cancer cells, previous studies have also reported comparable toxicity toward normal gastric mucosal cells (GES-1) [40]. It is important to note that CC_50_ and CC_25_ represent partially and minimally cytotoxic concentrations, respectively, and should not be described as completely non-cytotoxic. The potential contribution of cytotoxicity to the observed antiviral effects, particularly at CC_50_, cannot be entirely excluded. Nevertheless, the IC_50_ values for the suppression of EBV virion production (79.29 µM for AGS-EBV and 92.28 µM for HONE1-EBV cells) fell below the CC_25_ threshold in both cell lines, providing indirect evidence that antiviral activity is achievable at concentrations where cytotoxic effects are substantially limited. Accordingly, SI values of 3.60 and 3.26 in AGS-EBV and HONE1-EBV cells, respectively, are comparable to or exceed those reported for other natural EBV inhibitors in epithelial cancer cells, including luteolin (CC_50_ 68–272 µM) and emodin (CC_50_ 31–79 µM) [10,18]. Future studies should confirm these findings using concentrations below CC_10_, with parallel viability assessments and the normalization of viral readouts to cellular DNA content, to more rigorously distinguish antiviral effects from cytotoxic effects.

Capsaicin suppressed NaB/TPA-induced EBV lytic reactivation in EBV-positive GC and NPC cell lines by down-regulating EBV lytic gene expression at the mRNA level and inhibiting virion production. In addition, capsaicin also suppressed latent gene expression (specifically *LMP1*, *EBNA1*, and *EBER1*), demonstrating its capacity to interfere with both lytic activation and latent viral gene programs. These findings are consistent with previous work demonstrating the dual inhibitory effects of andrographolide on EBV lytic reactivation and cell survival in EBV-associated epithelial cancers, where mechanistic suppression of *BZLF1*, *BRLF1*, and *BALF5* occurred through the modulation of transcription factors, including MEF2D, SP1, and SP3, and epigenetic regulators [41,42,43]. Similarly, other natural compounds inhibit EBV reactivation through defined transcriptional regulatory pathways: luteolin disrupts SP1 binding to the BZLF1 and BRLF1 promoters [18,19], emodin represses Zp and Rp promoter activities through the down-regulation of SP1, with an IC_50_ of approximately 4.4 µM [10,44], and resveratrol suppresses NF-κB and AP-1 signaling in Burkitt lymphoma cells [17]. The flavonols kaempferol and apigenin further attenuate SP1 protein levels in NPC cells [45,46]. Collectively, capsaicin appears to exert a multilayered inhibitory effect on EBV infection by targeting multiple stages of the viral life cycle, consistent with the broader class of phytochemical EBV inhibitors. Capsaicin is also a naturally occurring dietary compound with an established safety profile and is already approved for topical clinical use, supporting its potential feasibility as a therapeutic candidate, although further in vivo pharmacokinetic and efficacy studies are required [30].

In the present study, proteomics revealed cell-type-dependent modulation of host cellular pathways underlying capsaicin-mediated EBV inhibition. In AGS-EBV cells, capsaicin induced substantial proteomic remodeling, with the enrichment of proteins associated with glycolysis, cytoplasmic translation, and heterochromatin formation, cellular states generally unfavorable for EBV lytic transcription, which requires an open chromatin configuration and active transcriptional machinery [47]. Downregulated proteins were enriched in pathways related to cytoskeletal organization, tight junctions, and motor proteins, which have previously been implicated in EBV lytic replication, virion trafficking, and virus-induced cellular remodeling [48,49]. In contrast, HONE1-EBV cells exhibited a markedly attenuated proteomic response, with DEPs primarily associated with chromatin-related biological processes, including telomere organization, nucleosome assembly, and chromatin organization, suggesting that capsaicin may suppress EBV lytic reactivation in NPC cells mainly through the reinforcement of chromatin structure rather than extensive metabolic remodeling. These differences likely reflect variations in EBV latency programs, epithelial origin, or basal chromatin states between NPC and GC cells, as well as possible differences in TRPV1 expression between the two cell lines, parameters not directly measured in this study and warranting future investigation. Notably, the enrichment of nucleosome assembly, telomere organization, and chromatin organization in capsaicin-treated HONE1-EBV cells is consistent with reports that chromatin-modulating compounds can stabilize EBV latency by restricting accessibility to viral immediate-early promoters [50,51]. Unlike histone deacetylase inhibitors, which may paradoxically promote EBV lytic gene expression, capsaicin did not induce pathways associated with transcriptional activation or DNA replication, further supporting its role as a suppressor of EBV lytic reactivation.

Metabolomic profiling revealed that capsaicin markedly perturbs metabolic pathways essential for EBV lytic reactivation, including purine and pyrimidine biosynthesis, polyamine metabolism, and glycolytic flux. These pathways are fundamental for viral DNA replication, protein synthesis, and virion assembly, indicating that capsaicin interferes with the metabolic requirements of productive EBV replication. Consistent with recent systems-level metabolomic and lipidomic analyses of lytic gamma-herpesvirus infection demonstrating that virion production is tightly dependent on the coordinated upregulation of glycolysis, nucleotide biosynthesis, glutaminolysis, and lipid metabolism [52], our findings support the concept that viral lytic replication is driven by extensive host metabolic rewiring. The convergence of aminoacyl-tRNA biosynthesis and purine metabolism in the integrated multi-omics analysis is consistent with previous reports showing that herpesvirus lytic replication is highly dependent on enhanced host translational capacity and nucleotide biosynthesis [17,52,53]. Furthermore, the involvement of glutathione-related pathways aligns with prior studies demonstrating that EBV lytic induction is regulated by cellular redox balance: elevated reactive oxygen species (ROS) promote EBV reactivation, whereas glutathione-dependent antioxidant systems counteract this process [54,55]. The observed cell-type-dependent differences in metabolic responsiveness are also consistent with prior reports showing that EBV-positive NPC cells exhibit stronger epigenetic and metabolic constraints than gastric epithelial models [56]. Collectively, these findings extend previous work by demonstrating that capsaicin targets multiple conserved metabolic vulnerabilities in EBV biology, particularly those governing translation, nucleotide biosynthesis, and redox regulation.

In this study, PPI network analysis identified HSP90AB1 (HSP90β) as the top downregulated hub protein in AGS-EBV cells and the primary molecular docking target among host chaperone proteins. This finding is consistent with biochemical evidence demonstrating that capsaicin binds to the N-terminal ATP-binding domain of HSP90, inhibits its ATPase activity, and promotes the lysosomal degradation of the co-chaperone HSP70 [57]. In the context of EBV biology, HSP90 stabilizes and facilitates the nuclear translocation of the EBV immediate-early transactivator Zta (BZLF1); therefore, disruption of Hsp90 chaperone activity represents a plausible, albeit preliminary, mechanistic basis for the suppression of EBV lytic gene expression through predicted interference with chaperone-mediated viral transcriptional activation, a hypothesis requiring experimental binding validation. Additionally, MYH9 and ANXA2 were identified as key downregulated hub proteins: MYH9 has been implicated in cytoskeletal remodeling and viral trafficking, and has been reported as a co-receptor facilitating the entry of SARS-CoV-2 and HSV-1 [58,59], whereas ANXA2 is involved in cytoskeletal organization, membrane trafficking, and viral replication across multiple herpesvirus species [60]. In HONE1-EBV cells, only a limited set of downregulated proteins was identified (MYL6, FSCN1, and YWHAZ); hence, the proteomic response in this cell line should be considered exploratory due to insufficient network complexity for robust topological analysis. The reduction in MYL6 may impair the intracellular dynamics required for the activation of EBV immediate-early genes, whereas YWHAZ downregulation may weaken the PKC and MAPK signaling networks required for viral reactivation [61,62,63,64,65].

Molecular docking simulations predicted that capsaicin may exhibit favorable binding affinities toward key host hub proteins (HSP90AB1, −7.9 kcal/mol; MYH9, −6.7 kcal/mol; ANXA2, −6.1 kcal/mol), upstream transcriptional regulators (RELA, −5.9 kcal/mol; STAT3, −5.7 kcal/mol; p53, −5.2 kcal/mol), and EBV lytic proteins (EA-D, −6.6 kcal/mol; BRLF1, −5.0 kcal/mol; BZLF1, −4.5 kcal/mol). These analyses are hypothesis-generating and exploratory; the predicted binding affinities have not been experimentally validated and should not be interpreted as evidence of direct physical interaction or biological activity. Experimental verification using biophysical binding assays, such as surface plasmon resonance (SPR), isothermal titration calorimetry (ITC), and microscale thermophoresis (MST), together with specificity profiling against irrelevant protein targets, will be required before firm conclusions regarding direct molecular interactions can be drawn. Taken together with the proteomic and metabolomic evidence, these computational findings provide preliminary support for a multilevel mechanism by which capsaicin may inhibit EBV lytic reactivation through the modulation of host signaling and metabolic pathways, as well as predicted interactions with viral lytic proteins.

Several limitations of the present study should be acknowledged. First, the findings are based on in vitro models, which may not fully reflect in vivo conditions, and the concentrations of capsaicin used may not be clinically achievable without targeted delivery systems. Second, the use of CC_25_ and CC_50_ as experimental concentrations means that a cytotoxic contribution to the observed reductions in viral readouts cannot be formally excluded, particularly at CC_50_; future studies should include concentrations below CC_10_ with parallel 48 h viability assessments and the normalization of viral readouts to cellular DNA content. Third, the substantially divergent proteomic responses between AGS-EBV and HONE1-EBV cells remain incompletely explained; differential TRPV1 expression, receptor density, and downstream signaling capacity between the two lines represent plausible alternative explanations that were not directly tested. Fourth, the key hub proteins identified were not functionally validated, the molecular docking results remain in silico predictions, and the identification of EBV-encoded proteins by proteomics was performed against a human database only, which requires validation against a virus-specific database. Fifth, the specificity of capsaicin-mediated suppression of EBV gene expression relative to global transcriptional activity was not directly measured; the inclusion of additional unrelated cellular gene markers or global RNA output measurements is recommended in future studies. Collectively, these considerations underscore the need for further mechanistic, in vivo, and pharmacological validation before clinical translation can be considered.

## 4. Materials and Methods

### 4.1. Reagents

Capsaicin was purchased from LGC Standards (Guildford, UK). The 12-*O*-tetradecanoylphorbol-13-acetate (TPA) and sodium butyrate (NaB) were obtained from Merck, Burlington, MA, USA).

### 4.2. Cell Lines and Culture Conditions

The EBV-positive GC cell line, AGS-EBV, and the EBV-positive NPC cell line, HONE1-EBV (kindly provided by Prof. Hironori Yoshiyama, Shimane University, Shimane, Japan), were cultured in RPMI-1640 medium (GIBCO, Grand Island, NY, USA) supplemented with 10% fetal bovine serum (FBS; GIBCO, Grand Island, NY, USA) and 1% penicillin-streptomycin mixture (GIBCO, Grand Island, NY, USA). Cells were maintained at 37 °C and in a humidified incubator with 5% CO_2_.

### 4.3. Evaluation of Cytotoxicity of Capsaicin

The cytotoxicity of capsaicin was evaluated using the SuperKine™ Maximum Sensitivity Cell Counting Kit-8 (CCK-8; Abbkine, GA, USA) assay. The AGS-EBV and HONE1-EBV cells were seeded in 96-well plates at a density of 5 × 10^3^ cells/well and incubated for overnight. Cells were then treated with or without capsaicin at various concentrations (0, 100, 200, 300, and 400 µM) and incubated for up to 96 h. Following incubation, 10 µL of CCK-8 reagent was added to each well, and plates were incubated for an additional 2 h. The absorbance was measured at 450 nm using an EnSight Multimode Plate Reader (PerkinElmer, Waltham, MA, USA). The concentration-dependent cytotoxic effects were expressed as the 50% cytotoxic concentration (CC_50_) and the 25% cytotoxic concentration (CC_25_).

### 4.4. Effect of Capsaicin Treatment on EBV Lytic Gene Expression

To investigate whether capsaicin inhibits EBV lytic reactivation, AGS-EBV and HONE1-EBV cells were seeded in 6-well plates at a density of 2.5 × 10^5^ cells/well and incubated overnight. Cells were pre-treated with or without capsaicin at CC_50_ and CC_25_ concentrations for 3 h, followed by induction of EBV lytic reactivation using TPA and NaB. Cells were then incubated for an additional 48 h. Following treatment, cells were harvested, and total RNA was extracted using TRIzol™ Reagent (Thermo Fisher Scientific, Waltham, Massachusetts, USA) according to the manufacturer’s instruction. The cDNA was synthesized by using the ReverTra Ace™ qPCR RT Master Mix kit (Toyobo, Osaka, Japan) according to the manufacturer’s instruction. Quantitative real-time PCR (qRT-PCR) was performed to determine the expression levels of EBV lytic genes, *BZLF1*, *BRLF1*, *BMRF1*, and *BLLF1*, and latent genes, *EBER1*, *LMP1* and *EBNA1*. Gene expression was analyzed using the comparative Ct method (2^−ΔΔCt^). Glyceraldehyde 3-phosphate dehydrogenase (*GAPDH*) and β-actin (*ACTB*) were used as internal controls. The primer sequences used in this study were adopted from our previous study and are listed in Table 5.

### 4.5. Quantification of EBV Genome Copy Number

To determine whether capsaicin inhibits the release of progeny virions, EBV genome copy numbers were quantified in the cultured supernatant collected 48 h post-treatment from cells treated with DMSO (vehicle control), capsaicin at CC_50_ and CC_25_, TPA/NaB, or a combination of capsaicin with TPA/NaB. The cell culture supernatant was harvested by centrifugation to remove cellular debris. Viral DNA was extracted using TRIzol™DNA extraction (Thermo Fisher Scientific, Waltham, MA, USA) according to the manufacturer’s instruction. The EBV genome copy number was then determined by quantitative PCR (qPCR) targeting the EBNA1 gene (Table 5). Absolute quantification was performed using a standard curve generated from known EBV copy numbers and copy numbers in the samples were calculated based on the corresponding Ct values and the linear regression equation. Results from three independent experiments were used to calculate the mean ± standard deviation (SD).

### 4.6. Evaluation of Selective Index of Capsaicin

To evaluate the antiviral efficacy and safety profile of capsaicin, its selectivity index (SI) was determined based on cytotoxicity and its inhibitory effect on EBV lytic reactivation. The inhibitory activity was assessed by quantifying the reduction in EBV genome copy number in capsaicin-treated cells, and the half-maximal inhibitory concentration (IC_50_) was defined as the concentration required to achieve a 50% reduction in viral copy number. The SI value was calculated as the ratio of the CC_50_ to the IC_50_, providing an estimate of the therapeutic window and safety profile of capsaicin.

### 4.7. Proteomics and Data Analysis

Cells were treated with DMSO (control), capsaicin, TPA/NaB, or a combination of TPA + NaB and capsaicin for 48 h, in three independent biological replicates (*n* = 3 per group). Total proteins were extracted in 50 mM ammonium bicarbonate, homogenized using a Mixer Mill 400 (Retsch, Germany), and centrifuged at 14,000 rpm for 5 min. Protein concentrations were determined by Bradford assay and normalized. Proteins were reduced with dithiothreitol (DTT), alkylated with iodoacetamide (IAA), and digested with trypsin at 37 °C overnight. The reaction was terminated with formic acid, and peptides were collected for liquid chromatography-mass spectrometry (LC-MS) analysis. Peptides were analyzed using an Agilent QTOF 6545XT LC-MS system with an Agilent Peptide Map column (Agilent technologies, Santa Clara, CA, USA) under a 0–90% acetonitrile gradient over 85 min. Raw data were processed using MaxQuant (v2.6.3) against the UniProt human and EBV databases available at https://www.uniprot.org/ (accessed on 20 December 2025), with a false discovery rate (FDR) of 1%. Protein quantification was performed using the MaxLFQ algorithm. Quality control metrics, including the number of unique peptides per protein (minimum two peptides required for quantification), were assessed to ensure data reliability.

The differentially expressed proteins (DEPs) were identified based on the criteria of log_2_ (fold change) ≥ 2 or ≤−2 and adjusted *p*-value ≤ 0.05 (Benjamini–Hochberg false discovery rate correction). The jvenn web tool available at https://jvenn.toulouse.inrae.fr (accessed on 23 December 2025 was used to categorize the DEPs. The functional annotation was performed using The Database for Annotation, Visualization, and Integrated Discovery (DAVID) available at https://davidbioinformatics.nih.gov/ (accessed on 23 December 2025) for gene ontology (GO) categories, including biological process (BP), cellular component (CC), and molecular function (MF), while pathway enrichment analysis was conducted using Kyoto Encyclopedia of Genes and Genome (KEGG). The top ten enriched terms were visualized using the ggplot2 package in RStudio (v4.3.3).

In addition, PPI networks were constructed using the STRING database (version 12.0) to investigate the functional interactions among DEPs. The list of DEPs was uploaded to the STRING database with Homo sapiens selected as the reference organism. Interaction networks were generated using a minimum required interaction score of 0.4 (medium confidence). The resulting interaction network data were subsequently imported into Cytoscape software (version 3.10.4) for visualization and network analysis. Key clusters within the PPI networks were identified using the molecular complex detection (MCODE) plugin in Cytoscape with the following parameters: degree cut-off = 2, maximum depth = 100, MCODE score > 5, and nodes ≥ 10. The top five significant PPI clusters were selected for further analysis [66]. Hub proteins were subsequently identified using Cytohubba plugin based on Degree, Maximum Clique Centrality (MCC), and Betweenness algorithms to determine proteins with high network connectivity and potential biological significance [67].

### 4.8. Metabolomics and Data Processing

Cells were seeded in 6-well plates and treated with capsaicin, TPA/NaB, or their combination for 48 h. Metabolites were extracted using pre-cooled methanol (1:3, sample:methanol), incubated at −20 °C, centrifuged, and the supernatants were dried, reconstituted in 0.1% formic acid, filtered, and transferred to LC vials. Samples were analyzed using a Dionex Ultimate 3000 HPLC system (Thermo Fisher Scientific, Sunnyvale, CA, USA) coupled to a Bruker compact QTOF mass spectrometer (Bruker, Billerica, MA, USA). Separation was performed on an Acclaim Advantage II C18 column under a gradient of water and acetonitrile. Data was acquired in both positive and negative ion modes over an *m*/*z* range of 50–1000. Raw data were processed using MetaboScape^®^ 2022 (Bruker, Billerica, MA, USA) with the T-ReX 3D workflow for peak detection and quantification. Metabolite identification was based on MS/MS spectra and retention time matching against the MetaboBase Personal Library 2.0. Processed data were exported and analyzed in Perseus (v1.6.8.0) using two-sample statistical testing, with *p*-values adjusted for multiple comparisons using the Benjamini–Hochberg method (adjusted *p* < 0.05). Metabolites with a variable importance in projection (VIP) score ≥ 1.0, derived from PLS-DA, were additionally used as a selection criterion for differential metabolites.

Multivariate and pathway analyses were performed using MetaboAnalyst 6.0 available at http://www.metaboanalyst.ca (accessed on 11 January 2026) to identify metabolic pathways associated with alterations induced under NaB/TPA-treated cells and co-treatment with capsaicin. The processed data were subjected to principal component analysis (PCA) to assess sample distribution and variability, and partial least-squares discriminant analysis (PLS-DA) to identify discriminative metabolites between groups. Model validity of PLS-DA was assessed by 5-fold cross-validation, with R^2^ and Q^2^ values reported to confirm model robustness and exclude overfitting. Differential metabolites were further analyzed using pathway enrichment and topology analysis to determine significantly affected metabolic pathways.

### 4.9. Molecular Docking

Molecular docking was performed to evaluate protein-ligand interaction, including binding affinity and ligand activity. The three-dimensional (3D) structure of capsaicin (ligand) was retrieved from PubChem database available at https://pubchem.ncbi.nlm.nih.gov/ (accessed on 25 January 2026) and energy-minimized and partial atomic charges were assigned using the Gasteiger method in AutoDock Vina (v1.5.6). Rotatable bonds were assigned automatically, and the ligand was saved in PDBQT format. The 3D structures of the candidate proteins (receptors), including MYH9 (9IHL), YWHAZ (1IB1), ANXA2 (2HYU), HSP90AB1 (1UYM), MYL6 (2M0G), FSCN1 (1DFC), RELA (1NFI), STAT3 (6NJS), p53 (1A1U), AP1 (5VPE), HIF-1α (1L3E), SP1 (1SP1), BZLF1 (7NX5), BRLF1 (6NCA), EA-D (8ZCP) were obtained from the Protein Data Bank (PDB) available at https://www.rcsb.org/ (accessed on 25 January 2026). Heteroatoms were removed, and the protein structures were protonated using BIOVIA Discovery Studio 2021. The binding pockets of each protein were predicted using PrankWeb available at https://prankweb.cz/ (accessed on 25 January 2026). Grid boxes were centered on the predicted binding pockets with dimensions of 34 × 26 × 26 Å. Docking was performed using AutoDock Vina with an exhaustiveness of 8 and a maximum of 20 output poses. The top-ranked pose with the lowest binding free energy (kcal/mol) was selected for further analysis. Docking of capsaicin with target proteins was performed using AutoDock Vina. Protein–ligand interactions were further analyzed using BIOVIA Discovery Studio 2021 and visualized using PyMOL (v3.1.5.1).

These molecular docking analyses are hypothesis-generating and serve to prioritize candidate protein–ligand interactions for future investigation. The predicted binding affinities have not been experimentally validated; therefore, the results should not be interpreted as evidence of direct physical interaction or biological activity.

### 4.10. Statistical Analysis

The statistical analysis was performed using the GraphPad Prism (version 10.2.3) software. Data are presented as mean ± standard deviation (SD). Differences among multiple groups were analyzed using one-way analysis of variance (ANOVA) followed by Tukey’s post hoc test, while comparisons between two groups were performed using an independent samples *t*-test. A *p* value < 0.05 was considered statistically significant.

## 5. Conclusions

In conclusion, this study demonstrates that capsaicin effectively inhibits Epstein–Barr virus (EBV) lytic reactivation in EBV-associated epithelial cancer cell models, including AGS-EBV and HONE1-EBV cells. Importantly, capsaicin suppressed EBV immediate-early, early, and late lytic gene expression, resulting in reduced virion production while maintaining selective antiviral activity at sub-cytotoxic concentrations. Furthermore, integrated proteomic and metabolomic analyses revealed coordinated modulation of host metabolic, chromatin, cytoskeletal, and redox-related pathways essential for EBV lytic progression. In addition, network-based analyses and in silico molecular docking provided computational evidence for potential interactions with key host regulatory proteins and EBV lytic proteins involved in viral activation and replication, though these predicted interactions await experimental confirmation by biophysical methods such as SPR, ITC, or MST. Collectively, these findings indicate that capsaicin suppresses EBV lytic reactivation through multi-level disruption of host cellular networks and viral regulatory machinery, supporting its potential as a candidate therapeutic agent for EBV-associated epithelial malignancies, although further in vivo and pharmacological validation is required.

## Figures and Tables

**Table 1:** Cytotoxic concentrations (CC_50_ and CC_25_) of capsaicin in AGS, AGS-EBV, and HONE1-EBV cells.

Cells	Capsaicin Concentrations (µM)	
	CC_50_	CC_25_
AGS	279.52 ± 2.52	171.22 ± 10.19
AGS-EBV	284.96 ± 9.05	179.84 ± 6.09
HONE1-EBV	301.22 ± 1.76	188.14 ± 4.38

**Table 2:** The IC_50_ and SI values of capsaicin in AGS-EBV and HONE1-EBV cells.

Cells	IC_50_ (µM)	SI * (Unitless)
AGS-EBV	79.29 ± 3.56	3.60
HONE1-EBV	92.28 ± 4.24	3.26

* SI stands for selective index, calculated as CC_50_/IC_50_.

**Table 3:** Docking interactions of capsaicin with hub proteins in AGS-EBV and HONE1-EBV cells.

Cells	Receptor	Binding Affinity (kcal/mol)	Amino Acid Residues
AGS-EBV	HSP90AB1	−7.9	Trp162, Phe138, Leu107, Phe170, Phe22, Met98, Val150, Ile26
	MYH9	−6.7	Phe456, Glu457, Glu175, Leu476
	ANXA2	−6.1	Glu180, Glu218, Asp181, His223, Ala179, Ile185, Pro222
HONE1-EBV	MYL6	−6.5	Tyr89, Arg37, Pro43, Leu93, Leu149, Ile148, Phe144, Val145
	FSCN1	−5.8	Trp2340, Ile2338, Cys2305
	YWHAZ	−5.4	Arg41, Ile217, Leu172, Ile166, Phe117

**Table 4:** Docking interactions of capsaicin with transcriptional regulators and EBV lytic proteins.

Receptor	Binding Affinity (kcal/mol)	Amino Acid Residues
Upstream regulatory proteins		
RELA	−5.9	Arg124, Cys120, Arg133, Gln128, Ala129, Lys122, Lys37, Val121
STAT3	−5.7	Leu520, Asp502, Trp510, Ile522, Trp501, Leu525, Ala505, Tyr539
p53	−5.2	Asn45, Phe38
AP-1	−4.7	Arg259, Glu251, Ala255, Lys258, Lys254
HIF-1α	−4.7	Arg36, Leu28, Ala37, Leu29
SP1	−4.4	Ala4, Arg11, Met13, Phe12
EBV lytic proteins		
BZLF1	−4.5	Arg190, Arg183, Asn182, Ala191, Arg187, Arg179
BRLF1	−5.0	Thr208, Ser212, Leu227
EA-D	−6.6	Arg266, Asp154, Ser291, Leu156, Tyr219, Pro218, Ile293

**Table 5:** Primer sequences utilized in this study.

Gene Name	Forward (5′–3′)	Reverse (5′–3′)
qRT-PCR Primers		
*BRLF1*	GGCCCAAAAATTGCAGATGT	CCCACGGGCGAGAATG
*BZLF1*	TCCGACTGGGTCGTGGTT	GCTGCATAAGCTTGATAAGCATTC
*BMRF1*	GCCGTTGAGGCCCACGTTGT	TGGGAATGGCAGGCGAGGGT
*BLLF1*	GCCGTTGAGGCCCACGTTGT	TGGGAATGGCAGGCGAGGGT
*EBER1*	CTACGCTGCCCTAGAGGTTTT	CAGCTGGTACTTGACCGAAGA
*LMP1*	TCCAGAATTGACGGAAGAGGTT	GCCACCGTCTGTCATCGAA
*EBNA1*	GGTCGTGGACGTGGAGAAAA	GGTGGAGACCCGGATGATG
*GAPDH*	AATCCCATCACCATCTTCCA	TGGACTCCACGACGTACTCA
qPCR Primers		
*EBNA1*	GGAGCCTGACCTGTGATCGT	TAGGCCATTTCCAGGTCCTGTA
*ß-actin*	GCCATGGTTGTGCCATTACA	GGCCAGGTTCTCTTTTTATTTCTG

## Data Availability

The original contributions presented in this study are included in the article/Supplementary Material. Further inquiries can be directed to the corresponding author.

## Supplementary Materials

The following supporting information can be downloaded at: https://www.mdpi.com/article/10.3390/ijms27115146/s1.

## Abbreviations

BP	Biological process
CC	Cellular component
CI	Confidence Interval
DEPs	Differentially expressed proteins
DMSO	Dimethyl sulfoxide
EBV	Epstein–Barr virus
FBS	Fetal bovine serum
GC	Gastric cancer
GO	Gene ontology
MF	Molecular function
NaB	Sodium butyrate
NPC	Nasopharyngeal carcinoma
OD	Absorbance value
OR	Odds ratio
PCA	Principal component analysis
PLS-DA	Partial least squares discriminant analysis
PPI	Protein–protein interaction
SD	Standard deviation
SI	Selectivity index
TPA	12-*O*-tetradecanoyl-phorbol-1,3-acetate
