# Supplementary material for: Multi-Omics Profiling Reveals Capsaicin Suppresses EBV Lytic Reactivation in Epithelial Cancers by Targeting Viral and Host Regulatory Networks
Source: Int J Mol Sci. 2026 Jun 5;27(11):5146. doi: 10.3390/ijms27115146 (PMC13258557; doi:10.3390/ijms27115146)
Supplement: Supplementary file 1 [file ijms-27-05146-s001.zip › Table S3.pdf]

Table S3. Candidate proteins from up-regulated and down-regulated DEPs in HONE1-EBV cells.

Protein symbol	Protein name	Degree	MCC	BC
**Up-regulation**				
H4C6	Histone H4	5	6	23
HSPA9	Stress-70 protein	5	6	21
H2BC9	Histone H2B type 1-H	2	2	-
RPLP2	60S acidic ribosomal protein P2	2	2	-
H3C12	Histone H3.1	2	2	-
AHNAK	Neuroblast differentiation-associated protein	2	2	-
C1QBP	Complement component 1 Q subcomponent-binding protein	1	1	-
PDIA3	Protein disulfide-isomerase A3	1	1	-
TTN	Titin	-	-	-
**Down-regulation**				
MYL6	Myosin light polypeptide 6	-	-	-
YWHAZ	14-3-3 protein zeta/delta	-	-	-
FSCN1	Fascin actin-bundling protein 1	-	-	-

* MCC: maximal clique centrality, BC: Betweenness centrality
