# Supplementary material for: Multi-Omics Profiling Reveals Capsaicin Suppresses EBV Lytic Reactivation in Epithelial Cancers by Targeting Viral and Host Regulatory Networks
Source: Int J Mol Sci. 2026 Jun 5;27(11):5146. doi: 10.3390/ijms27115146 (PMC13258557; doi:10.3390/ijms27115146)
Supplement: Supplementary file 1 [file ijms-27-05146-s001.zip › Table S1.pdf]

Table S1. PLS-DA cross-validation details show the R^2/Q^2 values obtained from PLS-DA method.

**Measure**	**1 comps**	**2 comps**	**3 comps**
**AGS-EBV: Negative mode**			
Accuracy	1.0	1.0	1.0
R^2	0.99631	0.99989	1.0
Q^2	0.94845	0.95266	0.95405
**AGS-EBV: Positive mode**			
Accuracy	1.0	1.0	1.0
R^2	0.99061	0.9997	0.99999
Q^2	0.82706	0.83473	0.83519
**HONE1-EBV: Negative mode**			
Accuracy	1.0	1.0	1.0
R^2	0.99583	0.99968	0.99999
Q^2	0.86094	0.87155	0.87116
**HONE1-EBV: Positive mode**			
Accuracy	1.0	1.0	1.0
R^2	0.99913	0.99999	1.0
Q^2	0.98741	0.98773	0.98776

*comps: components
