# Supplementary material for: Multi-Omics Profiling Reveals Capsaicin Suppresses EBV Lytic Reactivation in Epithelial Cancers by Targeting Viral and Host Regulatory Networks
Source: Int J Mol Sci. 2026 Jun 5;27(11):5146. doi: 10.3390/ijms27115146 (PMC13258557; doi:10.3390/ijms27115146)
Supplement: Supplementary file 1 [file ijms-27-05146-s001.zip › Table S2.pdf]

Table S2. Candidate proteins from up-regulated and down-regulated DEPs in AGS-EBV cells.

Protein symbol	Protein name	Degree	MCC	BC
**Up-regulation**				
TPI1	Triosephosphate isomerase	8	40	30.67
LDHA	Lactate dehydrogenase A	7	38	18.67
H6PD	GDH/6PGL endoplasmic bifunctional protein	6	36	5.33
PPIA	Peptidyl-prolyl cis-trans isomerase A	5	6	30.67
GAPDH	Glyceraldehyde-3-phosphate dehydrogenase	5	10	6
CS	Citrate synthase	4	24	-
PARP8	Protein mono-ADP-ribosyltransferase PARP8	4	7	13.67
H1-0	Histone H1.0	4	8	2
H2AX	Histone H2AX	4	8	2
SET	Protein SET	3	6	-
**Down-regulation**				
MYH9	Myosin-9	7	744	1.5
YWHAZ	14-3-3 protein zeta/delta	7	744	1.5
FLNA	Filamin-A	7	744	1.5
ANXA2	Annexin A2	6	720	-
HSP90AB1	Heat shock protein HSP 90-beta	6	720	-
EZR	Ezrin	6	720	-
MYL12B	Myosin regulatory light chain 12B	4	24	-
GRIK2	Glutamate receptor ionotropic, kainate 2	4	4	1
CACNG8	Voltage-dependent calcium channel gamma-8 subunit	3	4	1
NECTIN3	Nectin-3	2	2	-

* MCC: maximal clique centrality, BC: Betweenness centrality
