# Supplementary figures and images for: Multi-Omics Profiling Reveals Capsaicin Suppresses EBV Lytic Reactivation in Epithelial Cancers by Targeting Viral and Host Regulatory Networks
Source: Int J Mol Sci. 2026 Jun 5;27(11):5146. doi: 10.3390/ijms27115146 (PMC13258557; doi:10.3390/ijms27115146)

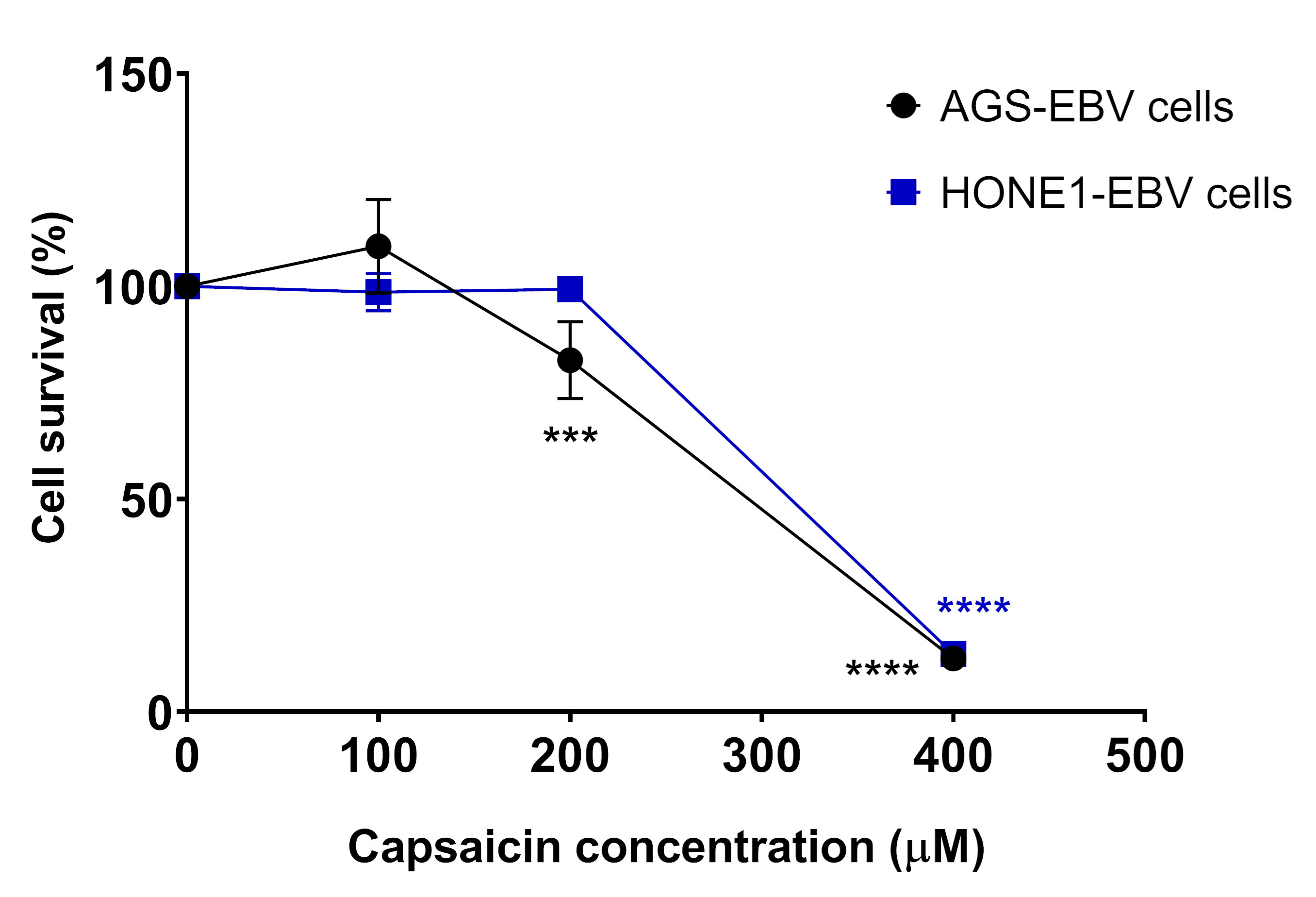

Supplement: Supplementary file 1 [file ijms-27-05146-s001.zip › Figure S1.jpg]

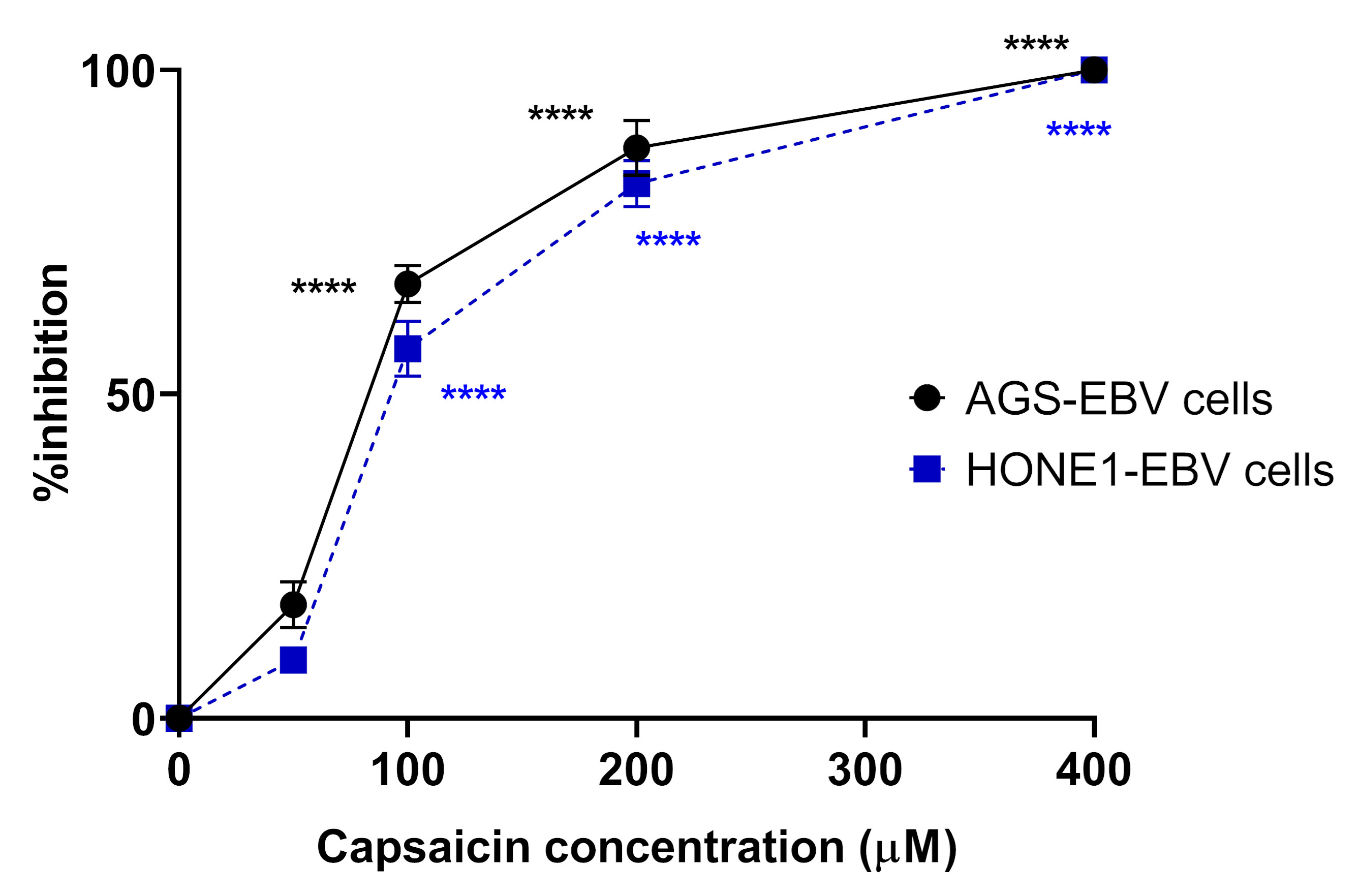

Supplement: Supplementary file 1 [file ijms-27-05146-s001.zip › Figure S2.jpg]
